# Postoperative Quality of Life in Patients with Hidradenitis Suppurativa Utilizing the Hidradenitis Suppurativa Burden of Disease Tool

**DOI:** 10.7759/cureus.13172

**Published:** 2021-02-06

**Authors:** Jiddu A Guart, Victor V Byers, Daniel C Sasson, Brian Bassiri-Tehrani, Matthew Ranzer, Chad Purnell

**Affiliations:** 1 Plastic Surgery, University of Illinois at Chicago, Chicago, USA; 2 Plastic Surgery, Northwestern University Feinberg School of Medicine, Chicago, USA

**Keywords:** hidradenitis suppurativa, quality of life, questionnaire, postoperative complications, hurley stage, hidradenitis suppurativa burden of disease (hsbod) tool, split thickness skin graft, secondary healing, complex closure

## Abstract

Background

Hidradenitis suppurativa (HS) severely impacts patients’ quality of life (QoL). Surgery has shown potential in improving a patient's QoL in severe disease. Previous studies have evaluated QoL after surgery, but lack a disease-specific questionnaire to better evaluate the unique burden of disease that patients with HS experience.

Objective

To measure postoperative QoL in patients with HS using a modified version of the disease-specific questionnaire, the Hidradenitis Suppurativa Burden of Disease (HSBOD) Tool.

Methods

A retrospective study was conducted using 19 patients who underwent surgery for HS. A demographic form and a 19-item disease-specific questionnaire were emailed to patients who consented to complete the survey. Patient-reported outcomes were recorded on a 0-100 scale (100 representing the highest burden of disease).

Results

Of the 24 patients that received the survey, 19 completed it in its entirety. The mean±SD Burden of Disease (BoD) score for each of the five domains assessed by the survey were: symptoms and feelings (62±27), daily activities (65±30), leisure (57±31), work and school (48±32), and personal relationships (56±27). Pearson’s correlation between the number of surgeries each patient underwent and their reported BoD scores were not significant. BoD scores were significantly higher in the symptoms and feelings domain for complex closure compared to both secondary intention and split-thickness skin grafting (STSG).

Conclusion

Despite having surgery, patients with hidradenitis still report impaired QoL. Further study is ongoing to determine how these measures compare to baseline preoperative values. This instrument provides a valuable tool to determine QoL in patients with hidradenitis.

## Introduction

Hidradenitis suppurativa (HS) is a chronic inflammatory skin disease that primarily affects the intertriginous areas of the body. Although the exact pathophysiology has yet to be elucidated, it is thought to be the result of an immune response following the obstruction of follicles within apocrine sweat glands [1,2]. Clinical manifestations include recurrent abscesses, sinus tract formation, scarring, and fibrosis. The characteristic pain, malodor, drainage, and chronicity of HS makes it one of the most physically and psychologically distressing dermatological diseases, impairing a patient’s quality of life (QoL) [3]. In fact, studies show that patients with HS have significantly higher rates of depression, anxiety, completed suicide, and low self-esteem than the general population [4]. Additional factors that negatively impact QoL include delays in diagnosis, sexual dysfunction, and chronic pain. Furthermore, there is an inverse relationship between QoL and Hurley HS severity staging, which ranges from stage I (mild disease) to stage III (severe disease) [5].

Surgery remains the treatment of choice for patients with severe disease, resulting in significant improvement in QoL, decreases in recurrence, and in some cases, remission of disease [6,7]. Recurrences may still occur in severe recalcitrant cases, and the disease burden is not always entirely eliminated. Therefore, improving QoL is a critical goal of surgery. A number of studies have been performed assessing the influence of various medical treatment modalities on patient QoL, but few with regards to surgery. Of the investigations that have been done, the generic Dermatology Life Quality Index (DLQI) was used [6,8]. Our group sought to offer insight into surgical outcomes for HS with a modification of the Hidradenitis Suppurativa Burden of Disease (HSBOD) tool [9]. The HSBOD tool has been shown to strongly correlate with the DLQI’s overall ability to assess QoL, while better assessing certain aspects of QoL that are uniquely affected in HS such as personal relationships and work/school activities. A secondary aim of this study was to compare various surgical treatment modalities and how these affect postoperative QoL.

## Materials and methods

A retrospective survey was administered to patients being treated for HS at a tertiary care center from April to June, 2020. Subjects were included if they underwent surgery for the treatment of their HS within the past 15 years by an American Board of Plastic Surgery-certified plastic surgeon and had completed surgical treatment. Subjects were excluded if they did not speak or read in English, were lost to followup, or were continuing to undergo or had planned surgical treatments for HS.

**Figure 1:**
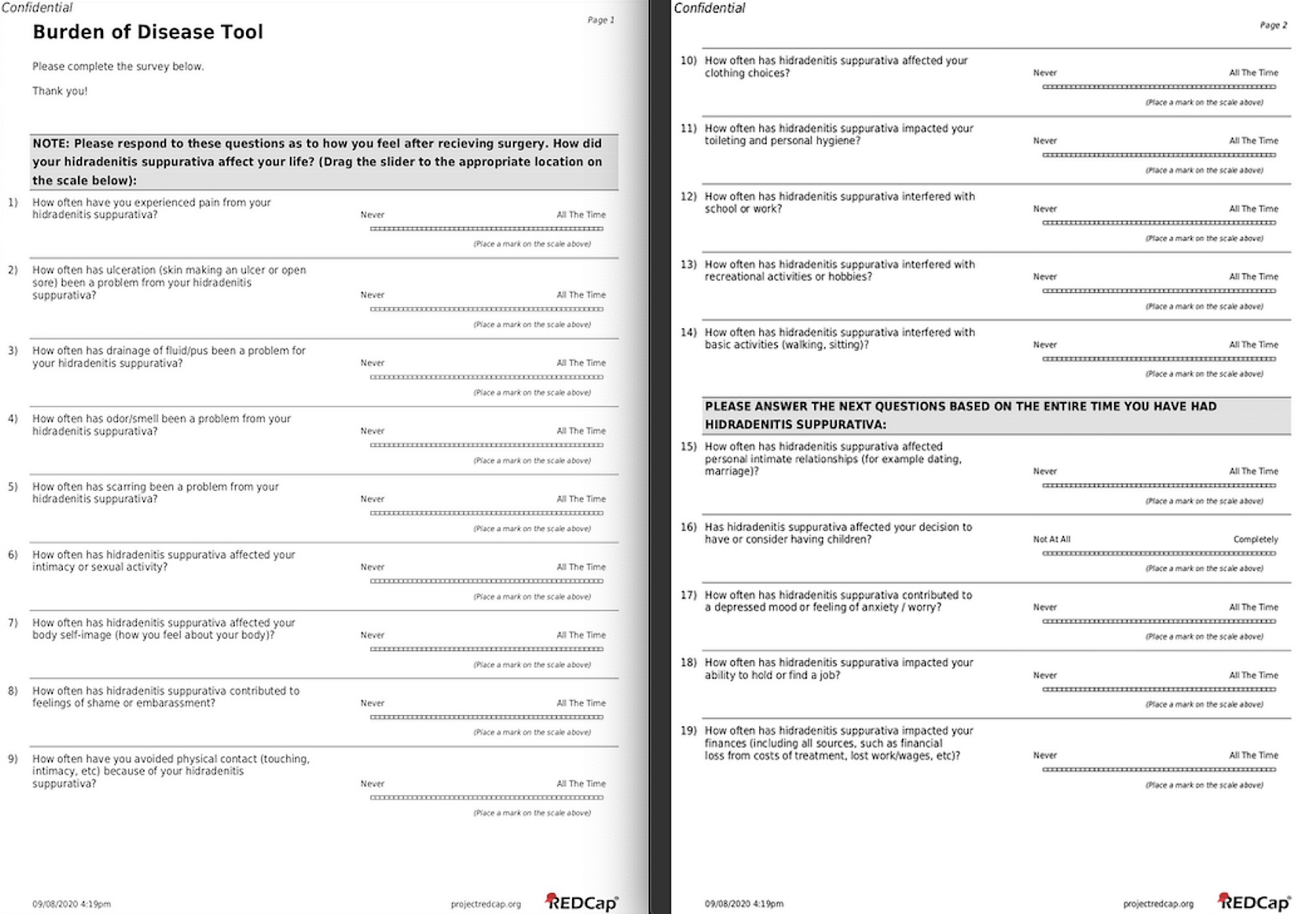
Modified Hidradenitis Suppurativa Burden of Disease Tool

The survey used in this study was the 19-question modified HSBOD (mHSBOD) tool with responses recorded using a visual analog scale (VAS) (Figure 1). Using an online data collection tool, Research Electronic Data Capture (REDCap), a demographics questionnaire and the mHSBOD survey were distributed via email, and patient responses were recorded. After the initial distribution of the survey, follow-up calls were made weekly over the span of three months and the survey was re-sent upon patient request.

Patient age, sex, race, annual income, tobacco use, alcohol use, and body mass index (BMI) were obtained via a demographic survey. Hurley stage for each patient was assessed using information from physical exams and direct classification by their dermatologists and plastic surgeons found in the electronic medical record. Additionally, the following data were collected: number of operations received by each patient, number of surgical sites treated, surgical site locations, types of wound closure, types of dressing used, follow-up intervals, time to wound closure (TTWC), recurrence rates and their management, and complications. Each patient was treated with wide excision of the diseased tissues with varying types of closure techniques, including complex closure, healing by secondary intention, or delayed split-thickness skin grafting (STSG).

In its original description, the first 14 questions of the HSBOD tool were used to measure QoL in the last four weeks. However, in order to better evaluate surgery’s impact on QoL, we adapted the first 14 questions of the HSBOD to assess patient QoL following surgery and kept the final five questions as assessment of lifetime impact on QoL, thereby creating the aforementioned mHSBOD (Figure 1). Patients scored each item on a VAS from 0, meaning no burden of disease (BoD), to 100, meaning maximum BoD. BoD was assessed across 5 QoL domains using the respective questions as follows: symptoms and feelings (questions 1-9 and 17), daily activities (questions 10 and 13), leisure (questions 11, 13, and 14), work/school (questions 12, 18, and 19), and personal relationships (questions 6-9, 15, and 16).

Descriptive statistics were used to evaluate the study data. Pearson’s correlation between the number of surgeries each patient individually received and their reported BoD scores for each of the 5 domains were measured. Pearson’s correlation was measured between the number of surgical sites per patient and the patient reported BoD score for each of the 5 domains. A Mann-Whitney U test was used to compare patient reported BoD scores in each domain by differences in treatment.

## Results

Of the 24 surveys distributed, 19 were completed in full, yielding a response rate of 79%. Participants had a mean ± SD age of 41 ± 14 years and the majority of respondents were female (15/24), mean BMI was 37 ± 10 and all patients were Hurley stage 2 or greater with the majority being stage 3 (17/24) (Table 1).

**Table 1:** Patient Characteristics

	N=19
Age (years ± SD)	41.105 ± 13.58
Gender	
Male (%)	4 (21%)
Female (%)	15 (79%)
Body Mass Index (kg/m2)	36.985 ± 9.4171
Smoking status	
Current smoker	3 (15%)
Former smoker	4 (21%)
Never smoked	12 (63%)
Hurley stage distribution	
II	2 (10%)
III	17 (90%)

An overview of surgical outcomes are shown in Tables 2-3. The majority of surgical sites were the axilla (n=21, 34.4%) followed by the groin (n=17, 27.9%). Of the total number of surgical sites, the majority were closed using complex closure technique (n=45, 61.6%). Of the 18 surgical sites that underwent healing by secondary intention (24.7%), 17 used a betadine wet-to-dry dressing (44.7%), followed by calcium alginate (n=9, 23.7). The mean follow-up length was 15 ± 23 months. Twelve surgical sites had recurrence and 11 of these were managed by a return to the operating room (RTOR). Of the complications that occurred, wound dehiscence in patients that underwent complex closure was the most common (n=24, 28.6%) followed by bleeding complications, which were controlled with bedside maneuvers (i.e. suture ligation, cautery, point pressure). There was no significant difference in complication rates between complex closure and secondary intention (P=0.117).

**Table 2:** Surgical Location and Methods Number of diseased sites for each patient, location of surgical sites, type of closure technique, and dressing types used in patients treated with secondary intention.

	Total # of patients (%) (N=19)
Number of diseased sites	
One	4 (21.1)
Two	5 (26.3)
Three	1 (5.3)
Four	4 (21.1)
Five	1 (5.3)
Six	2 (10.5)
Seven	1 (5.3)
Eight	1 (5.3)
	Total # of surgical sites (%) (N=61)
Surgical Sites	
Axilla	21 (34.4)
Groin	17 (27.9)
Other	9 (14.8)
Perineum	8 (13.1)
Breast/Chest	6 (9.8)
Type of Closure	
Primary closure	45 (65.2)
Secondary intention	18 (26.1)
Split thickness skin graft (STSG)	6 (8.7)
Dressing Type Used	(secondary intention only)
Betadine wet-to-dry	17 (44.7)
Calcium alginate	9 (23.7)
Saline wet-to-dry	4 (10.5)
Silvadene	4 (10.5)
Bacitracin	4 (10.5)

**Table 3:** Complications and Recurrence: Complex Closure, Secondary Intention, and Split-thickness Skin Graft Comparison of recurrence and complication data for each closure technique. Comparison of time to wound closure (TTWC) between complex closure and split-thickness skin graft (STSG).

	Complex Closure	Secondary Intention	STSG	Total
Total # of surgical sites	45	18	6	69
Recurrence				
Total (rate)	9 (20)	3 (16.7)	0 (0)	12 (17.4)
Timeframe: index operation to recurrence (months)	15.3 ± 11.7	11.7 ± 2.6	-	-
Management of recurrence				
Silver nitrate	1 (2.2)	0 (0)	0 (0)	1 (1.4)
Return to Operating Room	8 (17.7)	3 (16.7)	0 (0)	11 (15.9)
Complications				
Minor Bleeding	20 (44.4)	9 (50)	1 (16.6)	30 (43.4)
RTOR	6 (13.3)	6 (33.3)	0 (0)	12 (17.4)
Transfusion	3 (6.7)	4 (22.2)	0 (0)	7 (10.1)
Infection	7 (15.6)	5 (27.8)	0 (0)	12 (17.4)
Wound dehiscence	23 (51.1)	0 (0)	1 (16.7)	24 (34.8)
Total	59 (131)*	24 (133)*	2 (33.3)	85 (123)*
Time to Wound Closure Mean (months) ± SD	3.92±5.56	-	12.38±12.90	-
* values greater than 100% because some sites had multiple complications				

**Figure 2:**
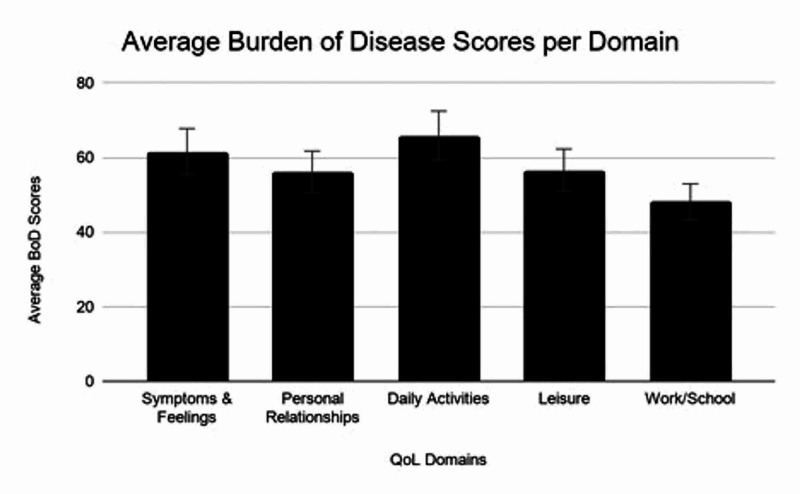
Average Burden of Disease Scores per Domain Average burden of disease (BoD) scores for all patients in each quality of life (QoL) domain.

The mean scores for each of the 5 domains were as follows: symptoms and feelings (62 ± 27), daily activities (65 ± 30), leisure (57 ± 31), work and school (48 ± 32), and personal relationships (56 ± 27) (Figure 2).

**Figure 3:**
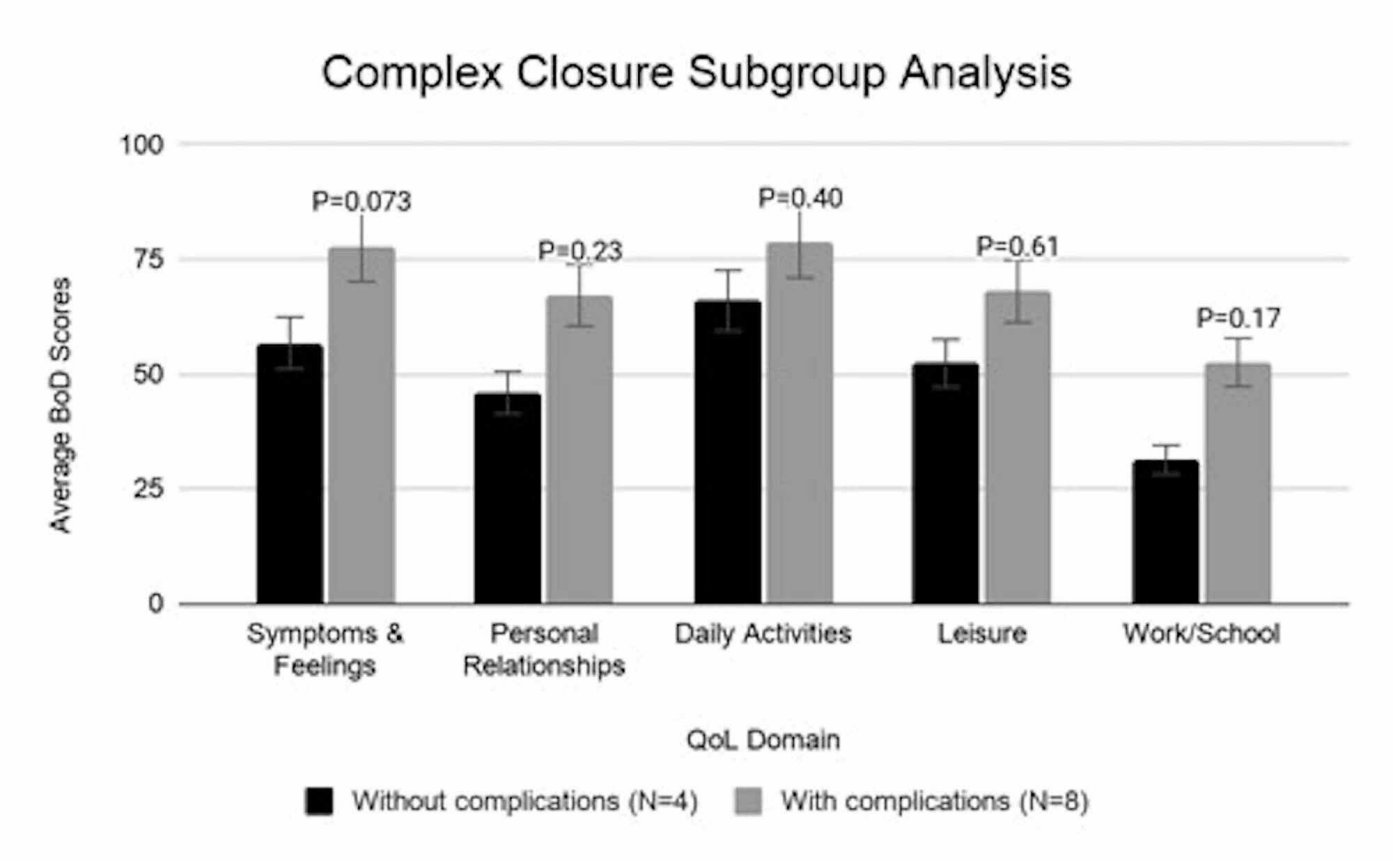
Complex Closure Subgroup Analysis Bar graph comparing average BoD scores in each domain between patients treated with complex closure without subsequent complications vs. complex closure with subsequent complications. Mann-Whitney U was performed and P-values are labeled above.

**Figure 4:**
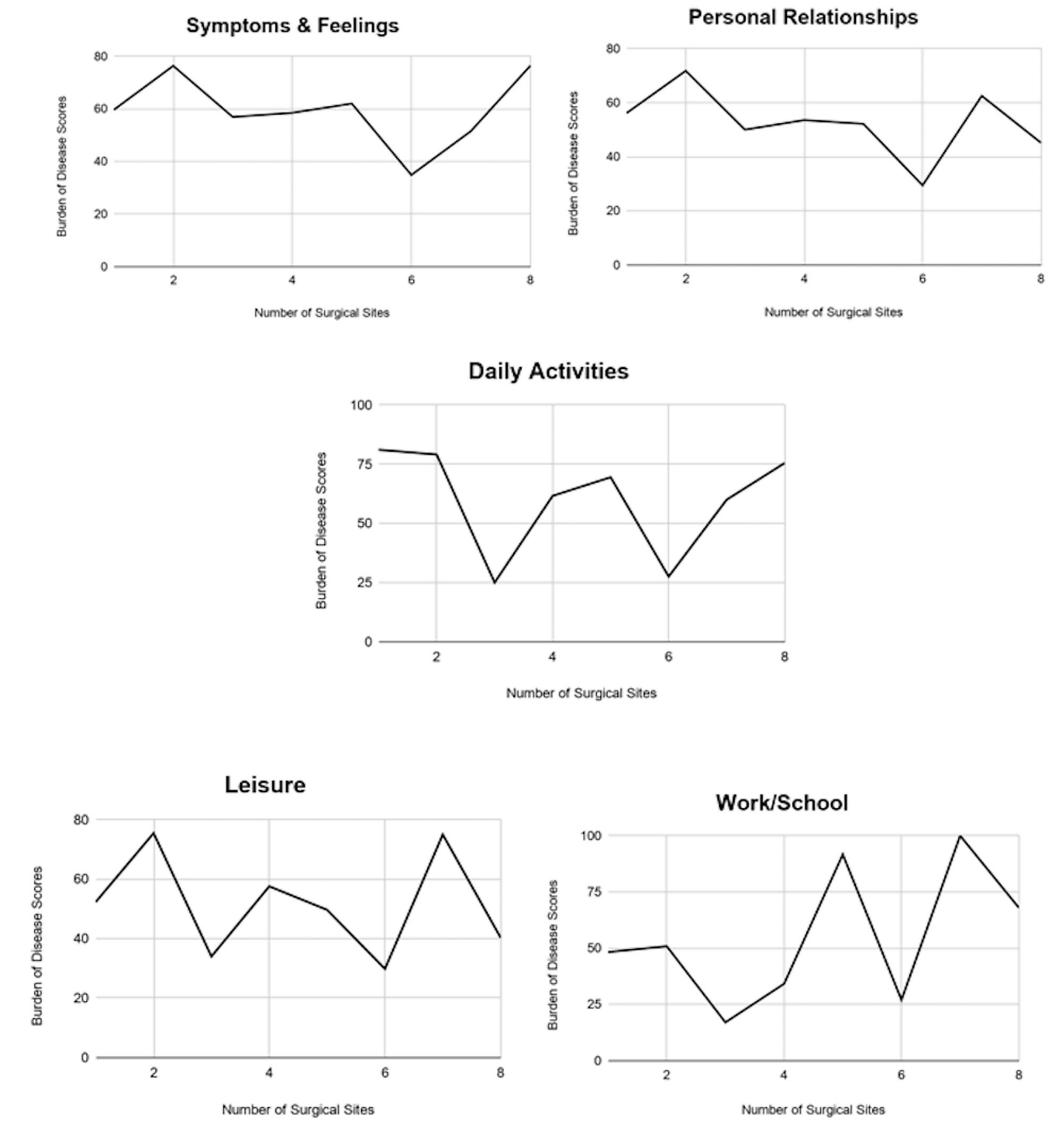
Relationship between Burden of Disease and Number of Surgical Sites Line graph depicting the relationship between burden of disease (BoD) scores and number of surgical sites for each of the five quality of life (QoL) domains.

Mean BoD scores for those who underwent complex closure were significantly higher than both secondary intention and STSG in the symptoms and feelings domain (70.88 ± 30.68 vs. 51.02 ± 38.13 and 32.45 ± 30.13 respectively, P = 0.035) (Table 4). To control for the higher rate of complications in the complex closure group, a subgroup analysis compared patients who underwent complex closure with or without complications and no significant difference was found (Figure 3). The mean TTWC for those that underwent complex closure was 3.92 ± 5.56 months and STSG was 12.38 ± 12.90 months (P = 0.118). Correlation of number of surgical sites with BoD scores in each domain were: symptoms & feelings (r=-0.18), personal relationships (r=-0.26), Daily activities (r=-0.34), leisure (r=-0.19), work/school (r= 0.15) (Figure 4).

**Table 4:** Closure Techniques and Burden of Disease Scores in each Quality of Life Domain Comparison of mean burden of disease (BoD) scores between closure techniques (complex closure, secondary intention, and STSG) in each of the five quality of life (QoL) domains.

	Complex Closure	Secondary Intention	STSG	
Total # of patients	12	5	2	
		Mean BoD Score ± SD		P^
QoL Domain				
Symptoms and Feelings	70.88±30.68	51.02±38.13	32.45±30.13	0.035
Personal Relationships	60.08±36.68	55.77±40.90	55.77±40.90	0.622
Daily Activities	74.58±29.66	55.40±46.49	39.75±40.19	0.343
Leisure	62.18±37.25	44.53±36.70	49.67±32.88	0.376
Work and School	45.47±36.93	52.27±42.16	53.83±50.71	0.925

**Figure 5:**
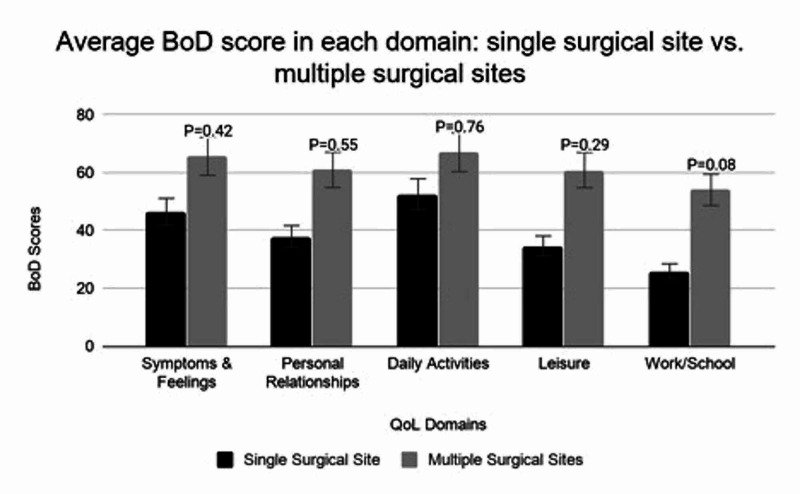
Average Burden of Disease Score for Each Domain: Single Surgical Site vs. Multiple Surgical Sites Bar graph depicting burden of disease (BoD) scores for a single surgical site vs multiple surgical sites. A Mann-Whitney U was performed and P-values are labeled above.

**Figure 6:**
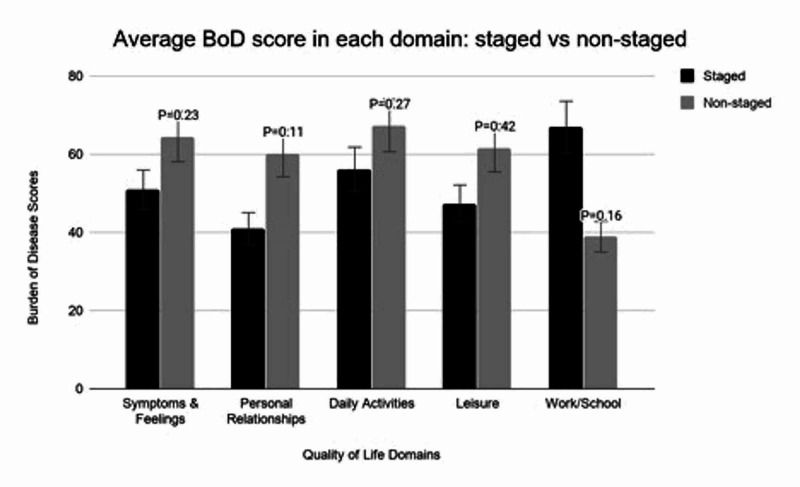
Average Burden of Disease Score in Each Domain: Staged vs. Non-Staged Bar graph comparing the BoD scores in the five QoL domains for patients undergoing staged vs non-staged procedures. A Mann-Whitney U was run to compare staged and non-staged procedures in each of the five domains, the P-values for each are depicted above.

We performed a Mann-Whitney U test to compare BoD scores for each of the five QoL domains between patients who underwent surgical treatment at a single site (n=4) vs. multiple sites (n=15). We found no significant difference between the mean BoD scores for those who had multiple sites vs. a single surgical site for all QoL domains (Figure 5). Comparison between staged and non-staged reconstruction resulted in non-significant differences between groups in all domains (Figure 6).

## Discussion

This retrospective cohort study utilized the mHSBOD questionnaire to study postoperative QoL in patients suffering from HS. Our study found that patients reported continued moderate BoD postoperatively, with scarring and self-image reported as most impacted domains. Additionally, we found no correlation between the number of individual surgeries received by each patient and their reported BoD.

Prens et al. found that patients with HS who were treated with surgery had DLQI scores which returned to baseline at their six-month follow-up interval, and Posch et al. found a significant decrease in the DLQI scores of patients treated with surgery after six months [6,10]. Extrapolating from this, one would assume that patients in our study would be at this improved level of QoL. However, given that preoperative values were not tested in this study, this cannot be necessarily assumed. Kouris et al. evaluated 22 unoperated patients with Hurley Stage III disease and found a mean DLQI of 19.32 [11]. In a different study, Prens et al. found that the mean post-operative DLQI score at six months was 15.6, with scores above 10 indicating severely impacted QoL.6 So perhaps the burden of disease is simply very high in a surgical HS population, and while surgery improves this, it does not resolve all of the QoL issues associated with the disease.

It follows that a moderate amount of impairment was found in every domain of the questionnaire. Beyond the possibilities discussed above, we feel there may be several reasons for this. Firstly, while all of the areas of surgical hidradenitis may have been treated for these patients, it is still possible that they struggle with continued lower-stage lesions that have an effect on QoL. Unfortunately, the study design does not have adequate granularity to capture the presence of these areas. Another possibility is that chronic HS results in long-lasting effects, even after the disease burden is decreased. The patients in our cohort have been affected by HS for over a decade on average, and therefore the lasting psychological impact may influence their perception of themselves and impact the scores reported, regardless of surgery [12]. Future studies are needed to evaluate the effects of multidisciplinary management in surgical HS, including psychotherapy [13,14]. Furthermore, scarring (item 5) and self-image (item 7) resulting from HS had the largest BoD postoperatively (74.84 and 70.85 respectively). Scarring is a permanent result of either surgery or HS and this BoD may likely be present regardless of surgical treatment.

In our patient group, recurrence occurred at 19% (n=12) of surgical sites. This is a lower recurrence rate compared to studies which used similar excision methods reporting recurrence rates between 27%-29% [15,16]. It is also possible that the patient definition of recurrence is different than the surgeons. Patients in this study were not re-examined after distribution of the survey unless it was part of routine follow up, and therefore potential mild recurrence may have been missed. In any case, we found that there was no significant correlation between the number of surgeries performed on each patient and the impact on their BoD scores. This is significant in that many patients with severe disease experience recurrence and undergo several operations. It seems from these data that once a surgical endpoint has been reached in HS, BoD is similar.

We found that in regards to symptoms and feelings, patients that treated with healing by secondary intention or STSG reported significantly lower BoD (P <0.05) compared with complex closure. This is somewhat counterintuitive - one might expect lower BoD scores in patients with complex closure due to the immediate and definitive closure of the wound at the time of the operation. Our findings could be due to the fact that HS has a predilection for intertriginous areas. These high-mobility areas predispose complex closures to complications such as dehiscence, limitation of range of motion, and scarring, possibly leading to lasting impacts on QoL [17]. Certainly, complication rates were higher amongst patients treated with complex closure in this cohort (70% versus 30% for secondary healing) In order to determine if the complications were the cause of increased BoD, a subgroup analysis was performed, which did not show significant differences amongst complex closure patients with and without a complication. However, this study is likely underpowered for this type of subgroup analysis. One would also think that increased time to wound closure would negatively affect QoL, but this did not seem to be correlated. Perhaps if the study timepoint would be during the healing process, wounds that took longer to close would cause a greater BoD, but once healed all wounds reach a similar endpoint. TTWC was still much shorter in patients who had a complex closure versus a skin graft or secondary intention healing, even with dehiscence complications.

This study illustrates the usefulness of the HSBOD tool to assess disease-related BoD in HS. Such research can open doors for clinicians to better guide treatment and form protocols, with superior evidence in regards to improving patient QoL. In addition to the disease-specific nature of the questionnaire, the short 19-item format is beneficial to the in-office practice of assessing patient QoL when compared to conventional, lengthier QoL assessment methods [8].

This study was limited by its retrospective nature, small sample size, and single time point of the survey. Given our small sample size, the comparisons between surgical techniques, single surgical site vs. multiple surgical sites, and staged vs. non-staged operations are almost certainly underpowered. We intend to expand this pilot study with a larger prospective study that follows patients throughout the surgical process. In addition, the lack of a preoperative baseline measurement to compare against postoperative QoL is a major limitation - it is possible that subjects in this study actually did have a significant improvement in QoL but started with very poor baseline scores [18]. Despite these limitations, our finding of moderately impacted BoD still remaining even after completion of surgical therapy is important for discussion with patients planning on undergoing surgery.

## Conclusions

Despite surgical intervention for HS, patient QoL is still impacted across the five domains assessed by the mHSBOD tool. The number of surgeries a patient receives does not have a significant correlation on their reported BoD. Patients who undergo complex closure for HS have a higher postoperative BoD than those treated with STSG or secondary intention healing. The mHSBOD tool is a useful tool to define the disease-specific effects of HS on QoL.
